# A Three-Dimensional Random Walk Algorithm for Estimating the Chloride Diffusivity of Concrete

**DOI:** 10.3390/ma13245700

**Published:** 2020-12-14

**Authors:** Hua Rong, Cong-Yan Zhang, Jian-Jun Zheng, Jian Zhang, Xin-Zhu Zhou, Bin Zeng

**Affiliations:** 1 Research Department of Science and Technology, Central Research Institute of Building and Construction, Metallurgical Group Corporation of China, Beijing 100088, China; ronghua@cribc.com (H.R.); zengbin@cribc.com (B.Z.); 2 Yuanpei College, Shaoxing University, Shaoxing 312000, China; CongyanZhang@usx.edu.cn; 3 School of Civil Engineering, Zhejiang University of Technology, Hangzhou 310023, China; jjzheng@zjut.edu.cn; 4 Jiyang College, Zhejiang Agriculture and Forestry University, Zhuji 311800, China; JianZhang_zjyc@163.com

**Keywords:** concrete, chloride diffusivity, EAM, RWA, spherical aggregate

## Abstract

The chloride diffusivity of concrete is an important parameter for assessing the long-term durability of coastal concrete structures. The purpose of this paper is to present a three-dimensional random walk algorithm (RWA) for estimating the chloride diffusivity of concrete. By analyzing the size distribution of aggregates, the equivalent interfacial transition zone (ITZ) thickness is derived in an analytical manner. Each aggregate is combined with the surrounding ITZ to construct an equivalent aggregate model (EAM) and the chloride diffusivity is formulated. It is found that the equivalent ITZ thickness decreases with the increase of practical ITZ thickness and aggregate volume fraction. The aggregate gradation influences the equivalent ITZ thickness to a certain extent. The relative chloride diffusivity of the equivalent aggregate is almost directly and inversely proportional to the equivalent ITZ thickness and the aggregate radius, respectively. The numerical results show that, when the EAM is adopted, the computational time is greatly reduced. With the EAM, concrete can be modeled as a two-phase material and the chloride diffusivity is estimated by applying the RWA. It is shown that, with the increase of mean square displacement and number of Brownian particles, the average chloride diffusivity of concrete approaches a stable value. Finally, through comparison with experimental data, the validation of the RWA is preliminarily verified.

## 1. Introduction

Reinforced concrete structures built in a chloride-laden environment often suffer from corrosion of reinforcement caused by penetration of chloride ions [1,2]. Many such engineering cases have been reported throughout the world [3,4,5]. Since the long-term performance of concrete infrastructure is greatly dependent on the diffusion rate of chloride ions in concrete, the chloride diffusivity plays a key part in designing and assessing coastal concrete structures [6,7].

In the past twenty years, a considerable number of experimental studies and theoretical analyses have been performed with a particular focus on the transport properties of concrete and on the key influential factors. By taking the water/cement ratio and the sand content as the primary control variables, three types of mortars were examined by a diffusion test and a migration test [6]. The test results showed that addition of sand into cement paste modifies the microstructure and chloride diffusivity of mortar. The tortuosity caused by sand particles exerts a more significant effect on the diffusion of chloride ions in mortar than the interfacial transition zone (ITZ). Using an accelerated method, Yang and Su quantified the effects of dilution, tortuosity, and ITZ on the chloride diffusivity of concrete based on the Bruggeman theory [8]. Caré [9] adopted a non-steady-state diffusion test to further analyze the relative importance between two competing factors: ITZ and tortuosity. In the theoretical aspect, a Padé approximation was proposed to estimate the chloride diffusivity of mortar [10]. An analytical solution was also presented for the chloride diffusivity of concrete with low aggregate volume fractions [11]. Caré and Herve proposed a composite sphere model for the chloride diffusivity of mortar [12]. The effects of various factors on the chloride diffusivity of concrete were comprehensively evaluated by a transfer matrix method with an inhomogeneous ITZ model [13]. It was also shown that aggregate shape influences the chloride diffusivity of concrete to a certain extent [14]. Besides a few analytical methods [15,16], the unit cell model is widely used, in which the volume ratios among various phase constituents are identical. In practical concrete, however, the ITZ thickness seems to be the same no matter how large the aggregate is [17]. As a consequence, a larger aggregate is of a smaller ITZ volume ratio in a unit cell. Thus, the prediction accuracy of the idealized unit cell with constant volume ratios will be affected to a certain extent. Although numerical methods can avoid this defect by simulating the heterogeneous structure of concrete, it is very time-consuming [18,19]. Therefore, it is still essential to develop a numerical method, with which the chloride diffusivity of concrete can be evaluated more reasonably.

This paper is aimed at developing a three-dimensional random walk algorithm (RWA) for estimating the chloride diffusivity of concrete. In the RWA, an equivalent aggregate model (EAM) is constructed to reduce the computational cost. Finally, the computational accuracy of the RWA is assessed through comparison with experimental results.

## 2. Simulation of Concrete Mesostructure

In what follows, only the steady-state diffusion of chloride ions in saturated concrete is considered. It is appreciated that transport of chloride ions in the pore solution is affected, to a certain extent, by the interactions among ions and the electrical double layer [20,21,22,23]. In view of the complexity of the two effects, they are neglected as a first step. To perform the random walk simulation, the concrete mesostructure needs to be reproduced as realistically as possible. For this purpose, a cubic element with a side length of a is selected. Aggregates are modeled as spherical and distributed at random within the element [24]. If the aggregate is divided into N grades [R_j_, R_j+1_] (j = 1, 2, …, N) and the volume percentage passing the sieve with radius R_j_ is P_v,j_, the approximate probability density function p_v_(R) with respect to the volume of aggregates is given by

(1) $$p_{v}\left(R\right)=\sum_{j=1}^{N}\frac{P_{v,j+1}-P_{v,j}}{R_{j+1}-R_{j}}[H(R-R_{j})-H(R-R_{j+1})],$$

where H(x) is the Heaviside step function and defined as

(2) $$H(x)=\{\begin{array}{ll} 1, & x>0\\ 0.5 & ,\;x=0\\ 0, & x<0 \end{array}.$$

The corresponding cumulative distribution function P_v_(R) is given by

$$P_{v}\left(R\right)=\int_{R_{1}}^{R}p_{v}(x)\mathrm{dx}$$

(3) $$=P_{v,k}+\frac{P_{v,k+1}-P_{v,k}}{R_{k+1}-R_{k}}(R-R_{k}),$$

where R_k_ ≤ R ≤ R_k+1_ and k = 1, 2, …, N. The number of aggregates per unit volume of aggregate can be formulated as

(4) $$N_{v}=\frac{3}{4\pi }\int_{R_{1}}^{R_{N+1}}\frac{p_{v}\left(x\right)}{x^{3}}\mathrm{dx}.$$

By substituting Equation (1) into Equation (4), one has

(5) $$N_{v}=\frac{3}{8\pi }\sum_{j=1}^{N}\frac{{(R}_{j}+R_{j+1}{)(P}_{v,j+1}-P_{v,j})}{R_{j}^{2}R_{j+1}^{2}},$$

It is easily shown that the probability density function p_n_(R) with respect to the number of aggregates is given by

(6) $$p_{n}\left(R\right)=\frac{3}{4\pi }\cdot \frac{p_{v}\left(R\right)}{N_{v}R^{3}}.$$

It follows from Equations (1) and (6) that

(7) $$p_{n}\left(R\right)=\frac{3}{4\pi }\sum_{j=1}^{N}\frac{{(P}_{v,j+1}-P_{v,j})}{N_{v}{(R}_{j+1}-R_{j}{)R}^{3}}[H(R-R_{j})-H(R-R_{j+1})].$$

The corresponding cumulative distribution function P_n_(R) is given by

$$P_{n}\left(R\right)=\int_{R_{1}}^{R}p_{n}(x)\mathrm{dx}$$

(8) $$=\frac{3}{8\pi }\sum_{j=1}^{k-1}\frac{{(R}_{j}+R_{j+1}{)(P}_{v,j+1}-P_{v,j})}{N_{v}R_{j}^{2}R_{j+1}^{2}}+\frac{{(R}^{2}-R_{k}^{2}{)(P}_{v,k+1}-P_{v,k})}{N_{v}{(R}_{k+1}-R_{k}{)R}_{k}^{2}R^{2}},$$

where R_k_ ≤ R ≤ R_k+1_ and k = 1, 2, …, N.

Knowing P_n_(R) and the aggregate volume fraction f_a_, the aggregates to be distributed within the cubic element can be generated [24]. These aggregates are placed into the element from largest to smallest and no overlap is permitted between them. During the placement process, periodic boundary conditions are imposed to eliminate artificial wall effects. Finally, the reconstruction of concrete mesostructure is completed once each aggregate is surrounded with an ITZ of thickness h.

## 3. Equivalent Aggregate Model and ITZ Thickness

It has been shown that there are two opposite effects on the diffusion of chloride ions in concrete. The dilution and tortuosity induced by aggregates decelerate the movement of chloride ions. On the other hand, the ITZ has a larger water/cement ratio (w/c) and higher porosity compared with the bulk cement paste. Furthermore, for a sufficiently high aggregate volume fraction, the ITZ percolates throughout the concrete specimen [25]. Thus, the ITZ and percolation effects accelerate the movement of chloride ions. Since the ITZ reduces the water/cement ratios of neighboring zones and the effect is internally balanced, its net effect on the movement of chloride ions is actually small [13]. To reduce the computational cost [26], an EAM is constructed as follows.

Since the microstructure of ITZ is different from that of bulk cement paste, it is more reasonable to model the ITZ as a distinct phase. Compared with the aggregate size, the ITZ thickness is usually much smaller [27]. As a result, when a Brownian particle walks near an ITZ, it will spend a great deal of time to walk even a short distance [18]. Therefore, the random walk simulation cannot efficiently be performed on the mesostructure of concrete.

**Figure 1:**
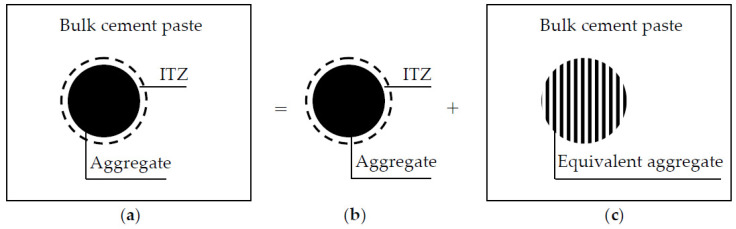
(**a**) Three-phase concrete; (**b**) equivalent aggregate; and (**c**) two-phase concrete.

To overcome this difficulty, an EAM is constructed by combining each aggregate with the surrounding ITZ, as shown in Figure 1. In practical concrete, when the surface-to-surface distance between two aggregates is smaller than twice ITZ thickness, their ITZ layers will overlap each other. As a result, the ITZ thickness h_eq_ in the EAM is smaller than the practical thickness h. To determine the equivalent ITZ thickness, the kth moment of area <R^k^> of p_n_(x) about the origin is defined as [28]

(9) $$\langle R^{k}\rangle =\int_{{\;R}_{1}}^{{\;R}_{N+1}}x^{k}p_{n}(x)\mathrm{dx}.$$

It is evident that <R>, 4π <R^2^>, and 4π <R^3^>/3 represent the average radius, surface area, and volume of spherical aggregates, respectively. By substituting Equation (7) into Equation (9), one has

(10) $$\langle R^{k}\rangle =\{\begin{array}{l} \frac{3}{4\pi }\sum_{j=1}^{N}\frac{{(P}_{v,j+1}-P_{v,j}{)\ln(R}_{j+1}{/R}_{j})}{N_{v}{(R}_{j+1}-R_{j})},\;\mathrm{for}\;k=2\\ \frac{3}{4\pi }\sum_{j=1}^{N}\frac{{(P}_{v,j+1}-P_{v,j}{)(R}_{j+1}^{k-2}-R_{j}^{k-2})}{(k-{2)N}_{v}{(R}_{j+1}-R_{j})},\;\mathrm{for}\;\mathrm{other}\;\mathrm{cases} \end{array}.$$

An algorithm was presented for evaluating the ITZ volume fraction f_i_ based on the statistical geometry of composites [11,29]. According to this algorithm, f_i_ is given by

(11) $$f_{i}=(1-f_{a})[1-\exp(-t_{1}h-t_{2}h^{2}-t_{3}h^{3})]$$

where the coefficients t_1_, t_2_, and t_3_ are expressed in terms of f_a_ and <R^k^> as [29]

(12a) $$t_{1}=\frac{{3f}_{a}\langle R^{2}\rangle }{(1-f_{a})\langle R^{3}\rangle }$$

(12b) $$t_{2}=\frac{{3f}_{a}\langle R\rangle }{(1-f_{a})\langle R^{3}\rangle }+\frac{{9f}_{a}^{2}{\langle R^{2}\rangle }^{2}}{2(1-f_{a}{)}^{2}{\langle R^{3}\rangle }^{2}}$$

(12c) $$t_{3}=\frac{f_{a}}{(1-f_{a})\langle R^{3}\rangle }+\frac{{3f}_{a}^{2}\langle R\rangle \langle R^{2}\rangle }{{(1-f_{a})}^{2}{\langle R^{3}\rangle }^{2}}+\frac{{\mathrm{wf}}_{a}^{3}{\langle R^{2}\rangle }^{3}}{{(1-f_{a})}^{3}{\langle R^{3}\rangle }^{3}}$$

with w being 0, 2, or 3. It was found that the effect of w on the computational accuracy is negligibly small but best results will be achieved for w=0 [11]. Thus, w is set to be zero in this paper. Since the ITZ volume fraction in the EAM should be equal to that in practical concrete, the equivalent ITZ thickness h_eq_ satisfies the following equation

(13) $$\frac{4\pi }{3}\int_{R_{1}}^{R_{N+1}}N_{v}f_{a}p_{n}{(x)(3x}^{2}h_{\mathrm{eq}}+{3\mathrm{xh}}_{\mathrm{eq}}^{2}+h_{\mathrm{eq}}^{3})\mathrm{dx}=f_{i}.$$

By substituting Equation (9) into Equation (13), one has

(14) $$N_{v}f_{a}{(h}_{\mathrm{eq}}^{3}+3\langle R\rangle h_{\mathrm{eq}}^{2}+3\langle R^{2}\rangle h_{\mathrm{eq}})=\frac{3}{4\pi }f_{i}.$$

Solving Equation (14) for h_eq_, one has

(15) $$h_{\mathrm{eq}}=\sqrt[{3}]{-\frac{q}{2}+\sqrt{\frac{q^{2}}{4}+\frac{p^{3}}{27}}}+\sqrt[{3}]{-\frac{q}{2}-\sqrt{\frac{q^{2}}{4}+\frac{p^{3}}{27}}}-\langle R\rangle$$

where p and q are equal to

(16a) $$p=3(\langle R^{2}\rangle -{\langle R\rangle }^{2})$$

(16b) $$q=2{\langle R\rangle }^{3}-3\langle R\rangle \langle R^{2}\rangle -\frac{3}{4\pi }\cdot \frac{f_{i}}{N_{v}f_{a}}.$$

**Figure 2:**
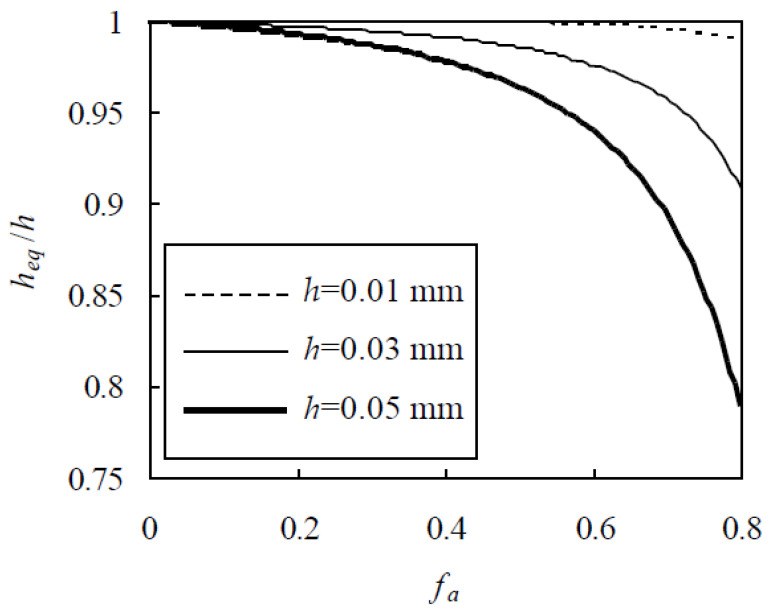
Effect of interfacial transition zone (ITZ) thickness on h_eq_/h.

It will be seen below that h_eq_ is closely related to the ITZ volume fraction in each equivalent aggregate and therefore the chloride diffusivity of concrete. It is interesting to investigate the effects of f_a_, h, and aggregate gradation on h_eq_, as seen from Equations (11), (15), and (16). To evaluate these influential factors in a quantitative manner, the Fuller gradation is first adopted with sizes from 0.15 to 16 mm. Thus, h_eq_/h is plotted against f_a_ in Figure 2 [24,30], which demonstrates that, for a small value of f_a_, h_eq_/h approaches unit and therefore ITZs seldom overlap. As f_a_ increases, more and more ITZs overlap and h_eq_/h decreases. For a given f_a_, h_eq_/h decreases with the increase of h. This is due to the fact that a larger h results in more overlaps of ITZs. For a given f_a_ at 0.6, 0.7, and 0.8, h_eq_/h at h = 0.05 mm is smaller than that at h = 0.01 mm by 5.76%, 10.1%, and 20.1%, respectively.

**Figure 3:**
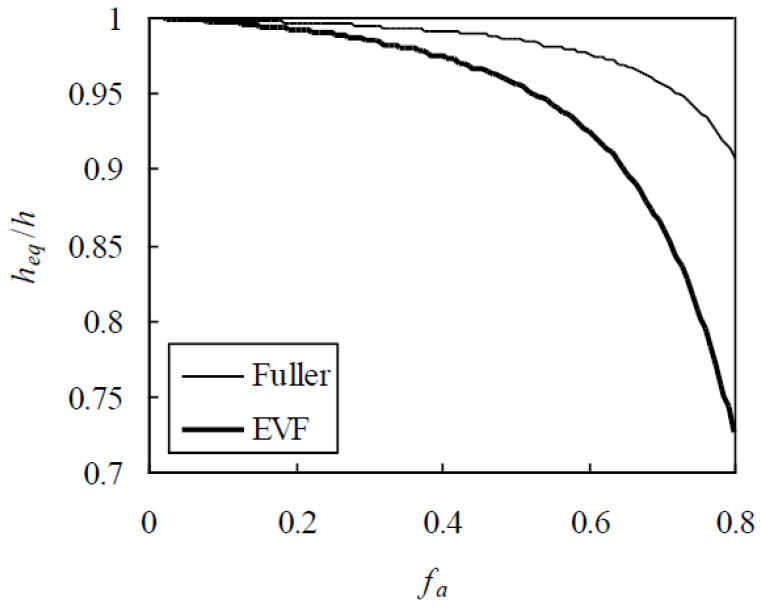
Effect of aggregate gradation on h_eq_/h.

Second, the effect of aggregate gradation on h_eq_/h is analyzed. For this purpose, two typical gradations, Fuller and equal volume fraction (EVF) [24,30], are considered and h = 0.03 mm. Thus, h_eq_/h is plotted against f_a_ in Figure 3, indicating that, for a given f_a_ at 0.6, 0.7, and 0.8, h_eq_/h for concrete with the EVF gradation is smaller than that with the Fuller one by 5.29%, 9.82%, and 20.1%, respectively. This attributes the fact that more small aggregates in the EVF gradation result in more overlaps of ITZs.

## 4. Chloride Diffusivity of Concrete

Duan et al. [31] proposed a completely explicit formula for the conductivity tensor of multi-phase media with various inclusions. From the formula, the chloride diffusivity of the equivalent aggregate shown in Figure 1 is given by

(17) $$D_{\mathrm{ea}}=D_{i}+D_{i}{\left[{\left(\frac{V_{a}}{V_{a}+V_{i}}\cdot \frac{D_{a}-D_{i}}{D_{i}+{(D}_{a}-D_{i})/3}\right)}^{-1}-\frac{1}{3}\right]}^{-1}$$

where D_a_ and D_i_ are the chloride diffusivities of aggregate and ITZ, respectively, and the aggregate volume V_a_ and the ITZ volume V_i_ are equal to

(18a) $$V_{a}=\frac{4\pi }{3}R^{3}$$

(18b) $$V_{i}=\frac{4\pi }{3}[(R+h_{\mathrm{eq}}{)}^{3}-R^{3}].$$

The chloride diffusivity of bulk cement paste is denoted by D_bcp_. Since D_a_ is much smaller than D_i_ and D_bcp_, D_a_ is set to be zero. Thus, Equation (17) becomes

(19) $$D_{\mathrm{ea}}=\frac{{2V}_{i}}{{3V}_{a}+{2V}_{i}}D_{i}.$$

By substituting Equations (18a) and (18b) into Equation (19), one has

(20) $$D_{\mathrm{ea}}=\frac{2[(R+h_{\mathrm{eq}}{)}^{3}-R^{3}]}{2(R+h_{\mathrm{eq}}{)}^{3}+R^{3}}D_{i}.$$

**Figure 4:**
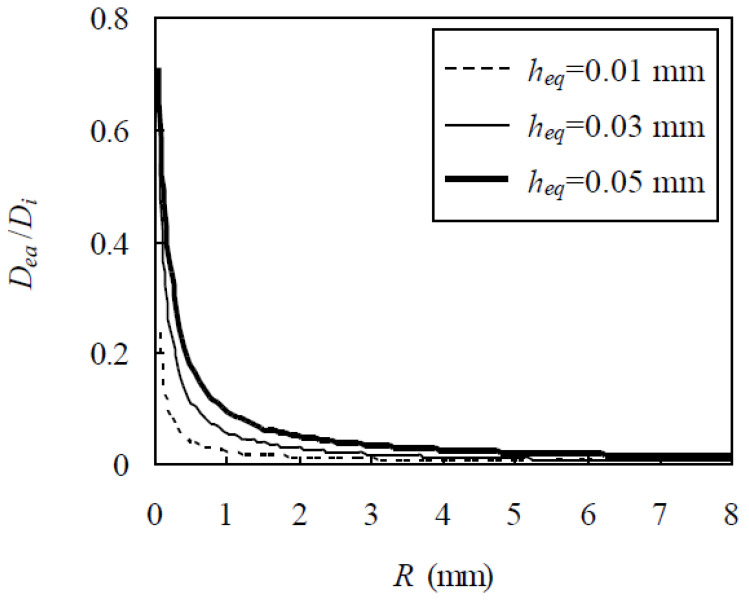
Relationship between D_ea_/D_i_ and R for different values of h_eq_.

It is seen from Equation (20) that, besides h_eq_, D_ea_/D_i_ is also dependent on the aggregate size. If R_1_ and R_N+1_ are set to be 0.075 and 8 mm, respectively, D_ea_/D_i_ is plotted against R in Figure 4, indicating that, since h_eq_ is much smaller than R, D_ea_/D_i_ is almost directly and inversely proportional to h_eq_ and R, respectively, as also seen from Equation (20).

With the EAM, concrete can be regarded as equivalent aggregates dispersed in a cement paste matrix. In this way, the RWA can directly be applied to the two-phase concrete [18,19].

**Figure 5:**
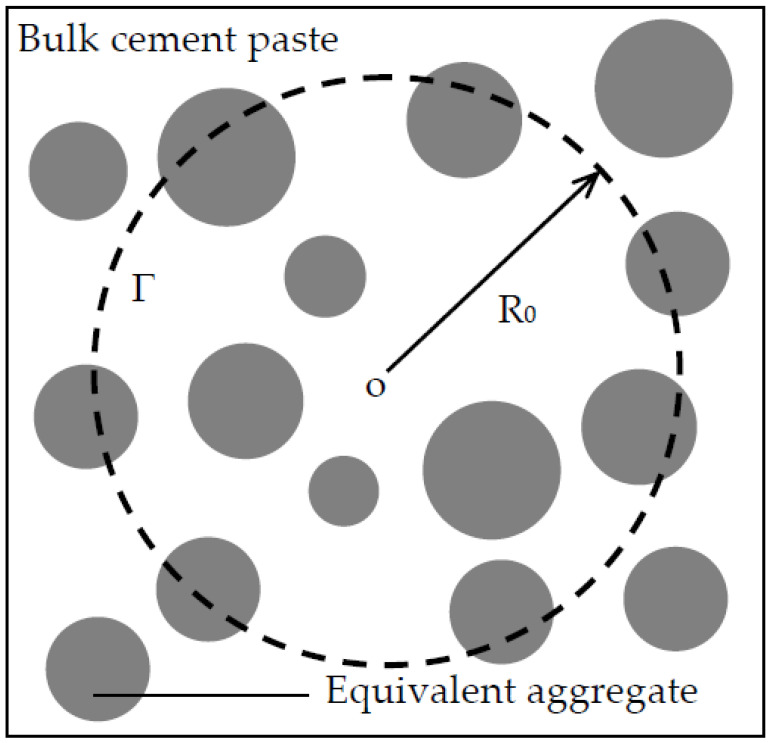
Two-phase concrete composed of bulk cement paste and equivalent aggregates.

In the beginning, a Brownian particle is randomly placed at a point, called the origin o, in concrete, as shown in Figure 5. Then, it walks step by step until hitting the spherical surface Γ of radius R_0_, i.e., the mean square displacement, centered at o for the first time. During the whole walk process, two cases should be considered separately. When the Brownian particle arrives at a point whose distance from any interfaces is larger than the prescribed value (=0.001 mm in this paper), a maximum sphere centered at the Brownian particle tangent to the interface is created. A random point is selected on the surface. If the spherical radius is r_i_ and the chloride diffusivity of the phase included in the sphere is D^(i)^, the mean time t(r_i_) for the Brownian particle to jump to the random point is [18]

(21) $${t(r}_{i})=\frac{r_{i}^{2}}{{6D}^{\left(i\right)}}.$$

It should be pointed out that Einstein and Smoluchowski derived an equation for Brownian movement in one dimension only under the assumption of Maxwell-Boltzmann distribution [32]. Later, the equation was extended to three dimensions. The theory of Brownian motion is usually developed for a system with no real boundaries. When the Einstein–Smoluchowski equation is directly applied to the diffusion of chloride ions in cementitious materials, the Brownian particle is restricted to three orthogonal directions [33]. When the Brownian particle encounters an impermeable solid phase, it is not permitted to step into the solid phase but the time spent is still counted for such an attempt [33]. There are two disadvantages of applying the Einstein–Smoluchowski equation to cementitious materials. First, this RWA simulates the detailed zigzag walk of the Brownian particle with small finite steps and is at least an order of magnitude slower than the Torquato and Kim one [34]. Second, when the Brownian particle comes near an interface between two permeable phases, it is difficult to compute the mean time and probability for it to cross the interface. To overcome these difficulties, Torquato and Kim [18] derived Equation (21) using the first passage time probability distribution. Based on Equation (21), the Brownian particle can walk directly to a random point on the surface of the maximum sphere, as stated above. Thus, there is no need to simulate the detailed zigzag walk of the Brownian particle with finite step sizes. Furthermore, when the Brownian particle is near an interface, they also formulated the mean time and probability for it to walk through the interface, as stated below.

**Figure 6:**
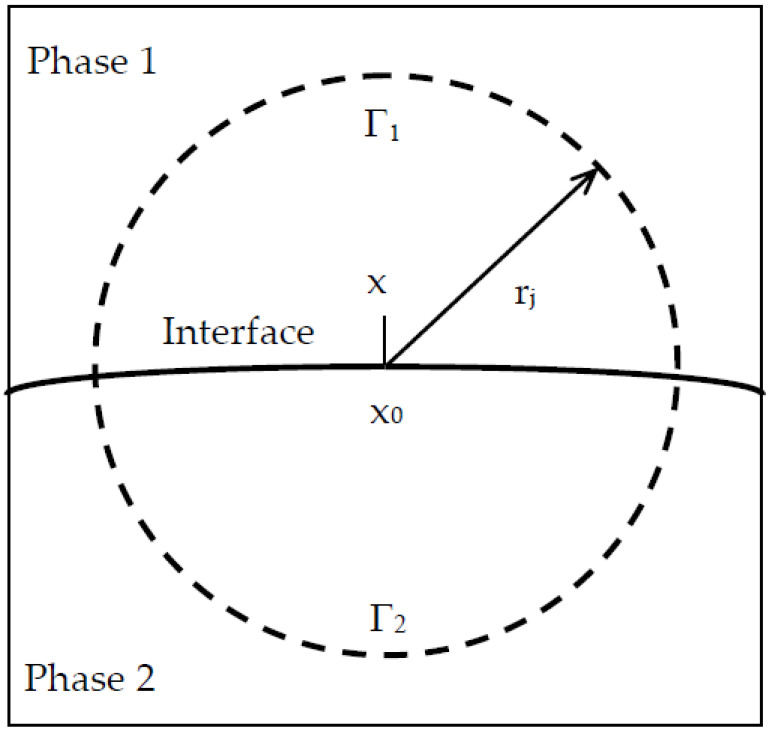
Brownian particle located near interface between phases 1 and 2.

When the Brownian particle arrived at a point x whose distance from an interface is smaller the prescribed value, the mean time can be evaluated as follows. If the projection of x on to the interface is denoted by x_0_, a sphere of radius r_j_ is then created centered at x_0_, as shown in Figure 6. The interface divides the sphere into two domains Ω^(1)^ and Ω^(2)^ with volumes V^(1)^ and V^(2)^ and chloride diffusivities D^(1)^ and D^(2)^ and the surface into two parts Γ^(1)^ and Γ^(2)^ with surface areas A^(1)^ and A^(2)^, respectively. The probabilities p_1_ and p_2_ for the Brownian particle to reach the surfaces Γ^(1)^ and Γ^(2)^ are equal to [18]

(22a) $$p_{1}=\frac{A^{\left(1\right)}D^{\left(1\right)}}{A^{\left(1\right)}D^{\left(1\right)}+A^{\left(2\right)}D^{\left(2\right)}}$$

(22b) $$p_{2}=\frac{A^{\left(2\right)}D^{\left(2\right)}}{A^{\left(1\right)}D^{\left(1\right)}+A^{\left(2\right)}D^{\left(2\right)}}$$

The mean time t(r_j_) for the Brownian to jump to a random point on the spherical surface is formulated as

(23) $${t(r}_{j})=\frac{{(V}^{\left(1\right)}+V^{\left(2\right)}{)r}_{j}^{2}}{{6(V}^{\left(1\right)}D^{\left(1\right)}+V^{\left(2\right)}D^{\left(2\right)})}.$$

It follows from Equations (21) and (23) that the mean time t(R_0_) is obtained as

(24) $${t(R}_{0})=\sum_{i}\frac{r_{i}^{2}}{{6D}^{\left(i\right)}}+\sum_{j}\frac{{(V}^{\left(1\right)}+V^{\left(2\right)}{)r}_{j}^{2}}{{6(V}^{\left(1\right)}D^{\left(1\right)}+V^{\left(2\right)}D^{\left(2\right)})}.$$

**Figure 7:**
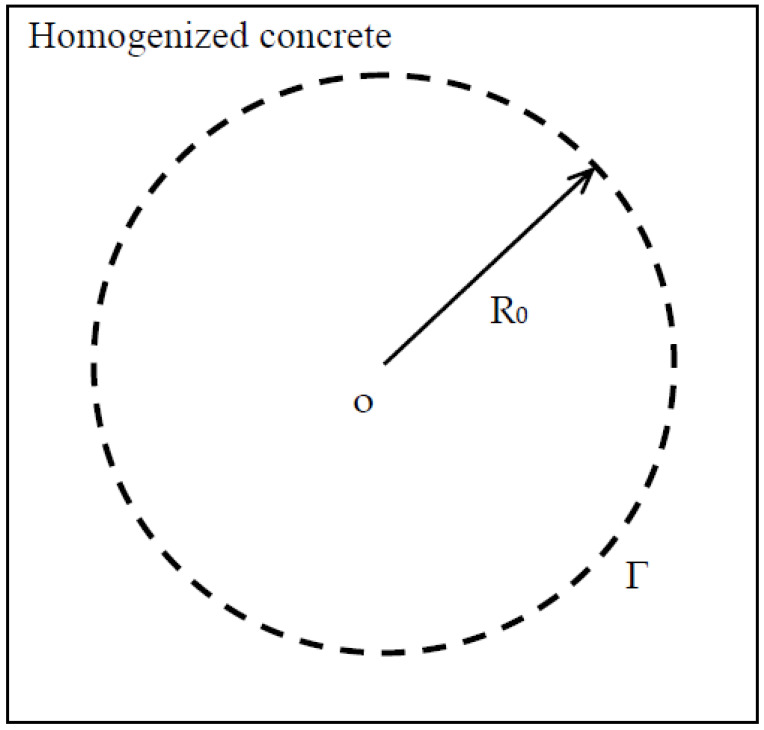
Homogenized concrete of chloride diffusivity D_con_.

On the other hand, when concrete is homogenized, it becomes a uniform medium of chloride diffusivity D_con_, as shown in Figure 7. Thus, for a sphere of radius R_0_, the mean time t(R_0_) for the Brownian particle to walk from the spherical center to a random point on the spherical surface is obtained as [18]

(25) $${t(R}_{0})=\frac{R_{0}^{2}}{{6D}_{\mathrm{con}}}.$$

From Equations (24) and (25), one can obtain D_con_ as

(26) $$D_{\mathrm{con}}=\frac{R_{0}^{2}}{\sum_{i}\frac{r_{i}^{2}}{D^{\left(i\right)}}+\sum_{j}\frac{{(V}^{\left(1\right)}+V^{\left(2\right)}{)r}_{j}^{2}}{V^{\left(1\right)}D^{\left(1\right)}+V^{\left(2\right)}D^{\left(2\right)}}}.$$

Based on the developed algorithm, a computer program is written with FORTRAN language for reconstructing the mesostructure of concrete and for implementing the random walk of Brownian particles.

**Figure 8:**
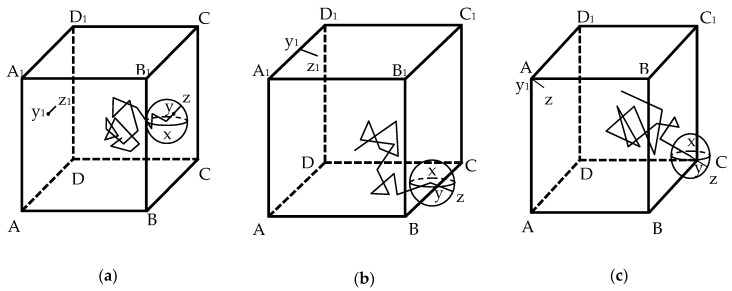
Brownian particle located close to (**a**) face; (**b**) edge; and (**c**) vertex.

**Figure 9:**
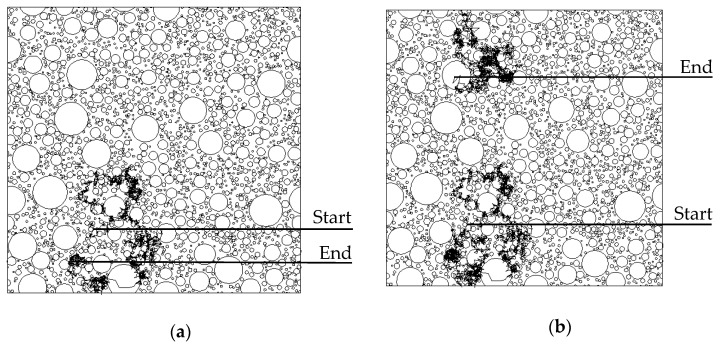

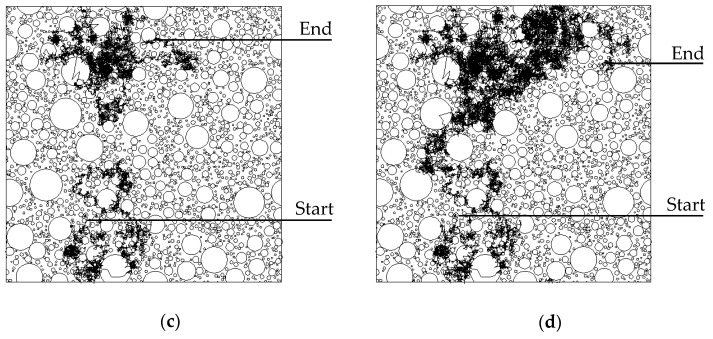
Random walk paths for (**a**) R_0_ = 20 mm; (**b**) R_0_ = 30 mm; (**c**) R_0_ = 40 mm; and (**d**) R_0_ = 60 mm.

During the whole random walk process, the Brownian particle possibly walks outside the cubic element. In such a situation, periodic boundary conditions are imposed. When it walks across the face BCC_1_B_1_ shown in Figure 8a, the edge BC shown in Figure 8b, or the vertex C shown in Figure 8c, the line segment yz, which extends beyond the element boundaries, will be reflected into the element on the opposite face ADD_1_A_1_, the edge A_1_D_1_, or the vertex A_1_, respectively, i.e., the line segment y_1_z_1_. When it walks across the other faces, edges, or vertexes, the procedure can be performed in a similar manner. To demonstrate the detailed walk process of a Brownian particle, a two-dimensional simulation square with a side of 60 mm is considered. The aggregate content is 0.5, h = 0.03 mm, and the Fuller gradation is adopted with sizes from 0.3 to 9.5 mm. The random walk paths are illustrated in Figure 9. Figure 9 shows that the Brownian particle first wanders in the lower left domain and then shifts to the upper left domain by crossing the bottom edge. After that, it explores the central domain for a while and finally comes near the upper right vertex.

**Figure 10:**
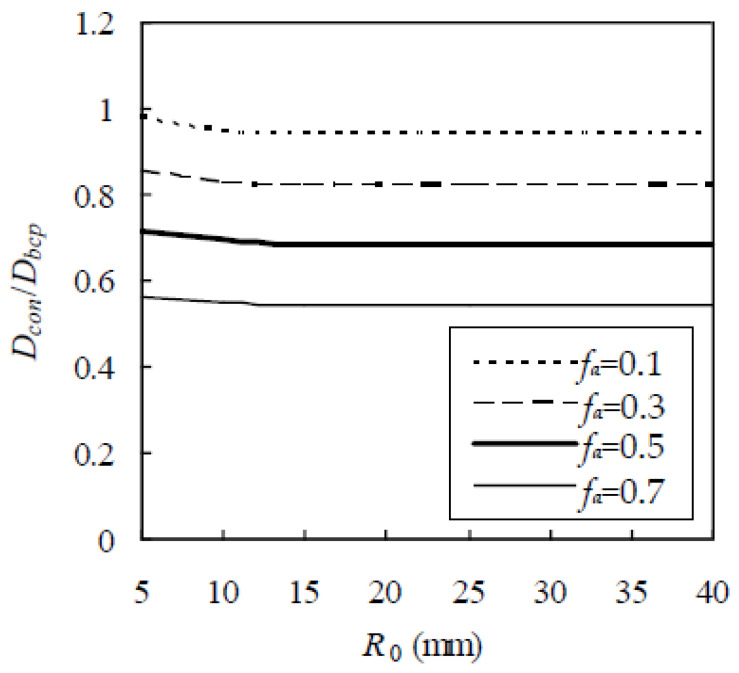
Effect of R_0_ on chloride diffusivity of concrete.

**Figure 11:**
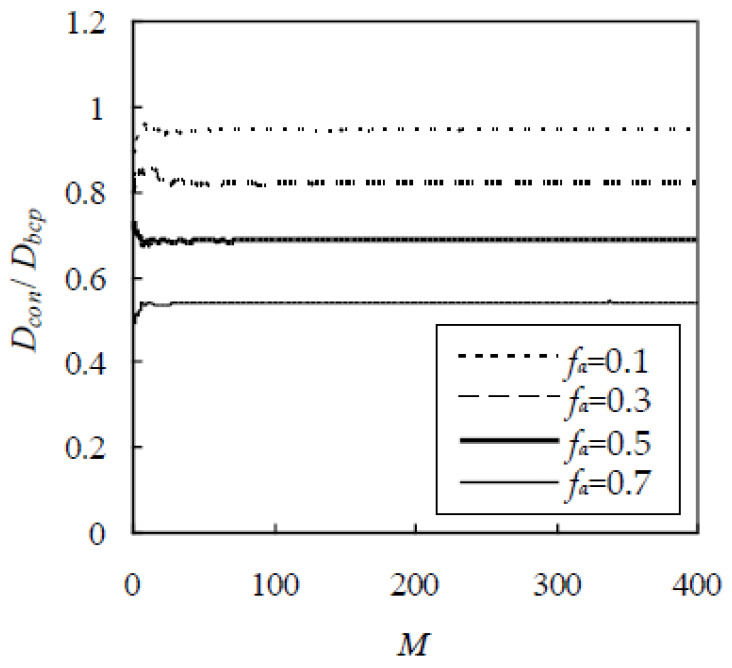
Effect of M on chloride diffusivity of concrete.

Before applying the RWA to concrete, a reasonable value of R_0_ needs to be known. Although a larger R_0_ results in a more accurate D_con_, a higher computational cost is required. In addition, the finite element size also leads to a slight fluctuation of D_con_ for different initial locations of the Brownian particle. The two shortcomings could be overcome by applying the ergodic hypothesis [35], i.e., D_con_ is taken as the average value over the random walks of M Brownian particles. For this purpose, a cubic element with a side of 20 mm is selected. The Fuller gradation is adopted with sizes from 0.3 to 9.5 mm, f_a_ is 0.1, 0.3, 0.5, and 0.7, and D_i_/D_bcp_ = 5. Thus, D_i_/D_bcp_ is plotted against R_0_ in Figure 10, which indicates that D_i_/D_bcp_ first decreases gradually with the increase of R_0_ and then keeps unchanged when R_0_ exceeds 20 mm. By taking R_0_ as 40 mm, D_i_/D_bcp_ is plotted against M in Figure 11, which indicates that D_i_/D_bcp_ fluctuates slightly for a smaller value of M. When M exceeds 250, D_i_/D_bcp_ keeps unchanged. The results are similar for other f_a_, D_i_/D_bcp_, and aggregate gradations. As a conservative estimate, R_0_ = 40 mm and M = 450 are adopted in this paper.

**Figure 12:**
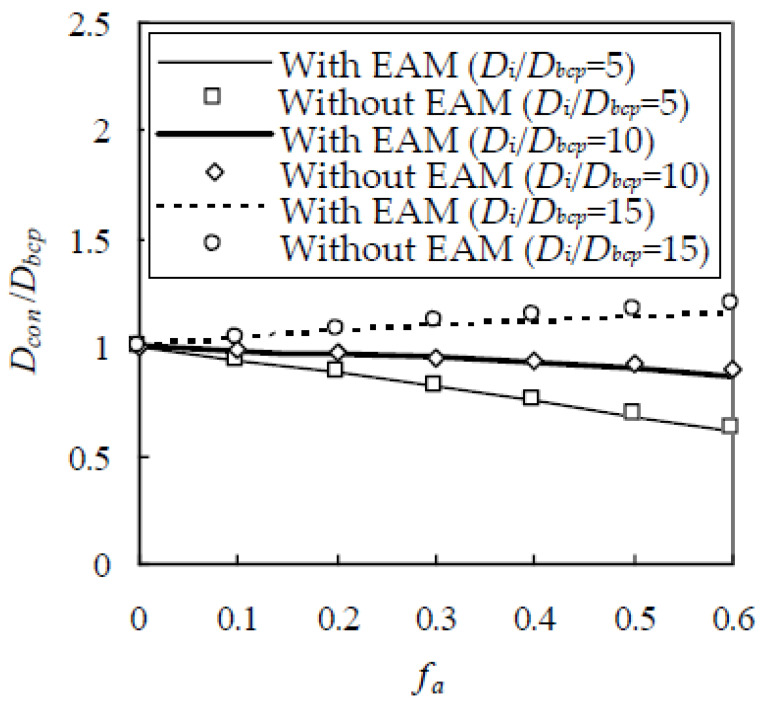
Comparison of estimated chloride diffusivity of concrete with and without equivalent aggregate model (EAM).

As stated in the previous section, the EAM is constructed in this paper to reduce computational cost. To show the validity of the EAM, a computer simulation is performed on a cubic element with a side length of 20 mm on a Thermaltake workstation. In the simulation, the Fuller gradation is adopted with sizes from 0.3 to 9.5 mm, f_a_ varies from 0.1 to 0.6, and D_i_/D_bcp_ is taken as 5, 10, and 15. The estimated D_i_/D_bcp_ is plotted in Figure 12, indicating that D_i_/D_bcp_ with EAM is very close to that without EAM. For a given value of D_i_/D_bcp_ at 5, 10, and 15, the average relative error between them is 0.858%, 1.88%, and 2.56%, respectively. However, the computational time is greatly reduced. For example, when the EAM is adopted, the computational time for concrete with f_a_ = 0.5 decreases by 72.6%. Therefore, the EAM gives similar results but at significantly reduced computational cost.

## 5. Experimental Verification

**Figure 13:**
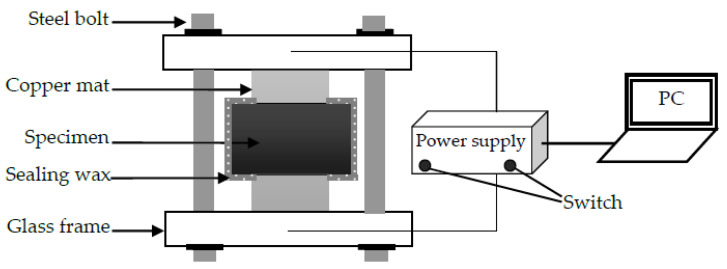
Schematic of test device.

To verify the validity of the developed RWA, a chloride diffusion test was performed. In the test, specimens with a w/c of 0.6 were cast with ordinary Portland cement. The Fuller gradation was adopted with sizes from 0.3 to 9.5 mm and f_a_ was 0.0, 0.15, 0.35, 0.55, and 0.75. After 24 h of casting, these specimens were demolded and cured in water at 21 °C for 28 days. The electrical conductivity method was adopted to measure D_con_ [36]. The test device is schematically shown in Figure 13.

**Figure 14:**
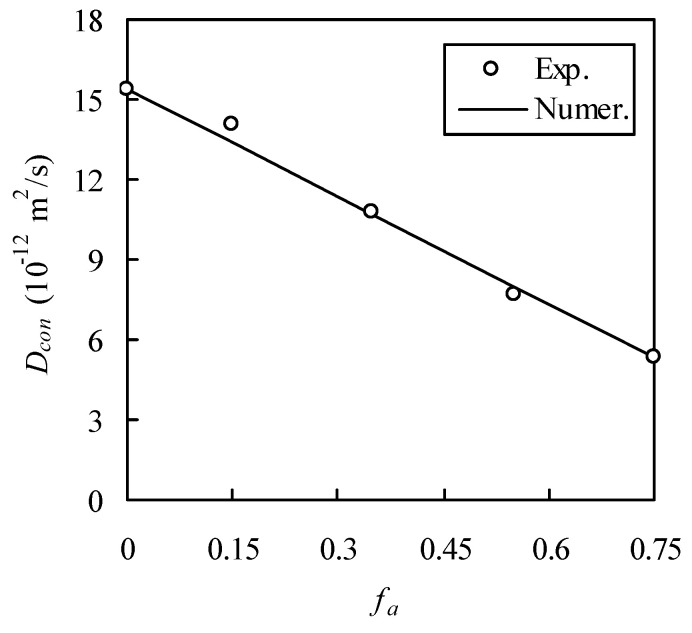
Comparison of numerical method with self-conducted experimental results.

Since h was not measured, it has to be estimated. It was confirmed that, for normal-strength concrete, h is in the range between 0.01 to 0.05 mm [27]. Thus, h is set to be 0.03 mm, i.e., the average between 0.01 and 0.05 mm. In this test, D_bcp_ (i.e., for concrete with zero aggregate volume fraction) was measured as 15.4 × 10^−12^ m^2^/s. At present, it is very tough to directly determine D_i_ in the laboratory, and therefore this paper resorts to experimental calibration. In this test, D_con_ at f_a_ = 0.75 was measured as 5.38 × 10^−12^ m^2^/s. Thus, D_i_ is obtained, by the inverse method, as 62.5 × 10^−12^ m^2^/s. With these parameters known, the random walk is performed to estimate D_con_, as shown in Figure 14, which shows a good agreement between the RWA and the measured D_con_. The relative error between them is 4.99%, 0.70%, and 3.25% for f_a_ = 0.15, 0.35, and 0.55, respectively.

**Figure 15:**
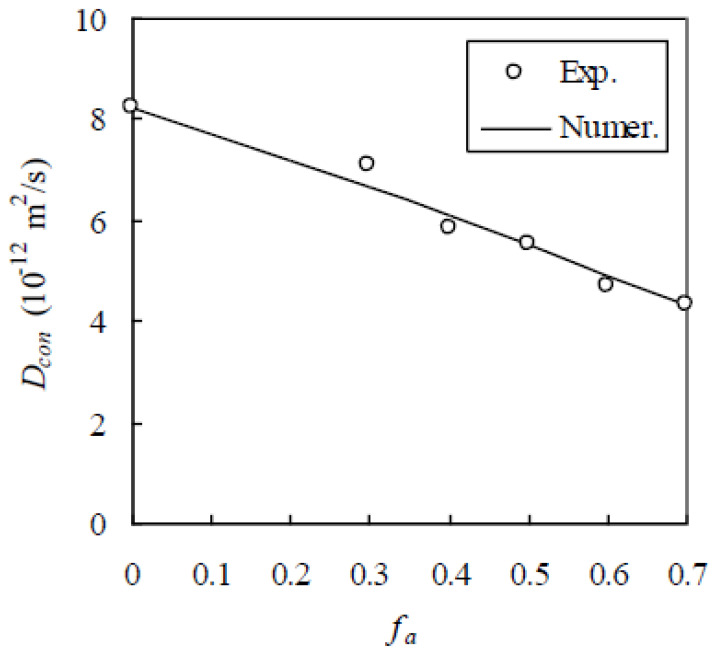
Comparison of numerical method with experimental results of Zheng and Zhou [37].

To further verify the RWA, the experimental data of Zheng and Zhou [37] are chosen. In their experiment, the cement type, aggregate gradation, accelerated method, and curing conditions are the same as those in the last experiment. But w/c was 0.5 and f_a_ was 0.0, 0.3, 0.4, 0.5, 0.6, and 0.7. The experimentally measured D_con_ was shown in Figure 15. Likewise, h = 0.03 mm. In the test, D_con_ was measured as 8.21 × 10^−12^ m^2^/s and 4.29 × 10^−12^ m^2^/s for f_a_ = 0.0 and 0.7, respectively. The former is D_bcp_, i.e., D_bcp_ = 8.21 × 10^−12^ m^2^/s and the latter is adopted to calibrate D_i_, which is obtained as 51.6 × 10^−12^ m^2^/s. Thus, D_con_ is computed by the RWA, as shown in Figure 15, indicating that the RWA agrees well with the experimental results. When f_a_ = 0.3, 0.4, 0.5, and 0.6, the relative error is 5.94%, 4.26%, 0.36%, and 4.08%, respectively.

**Figure 16:**
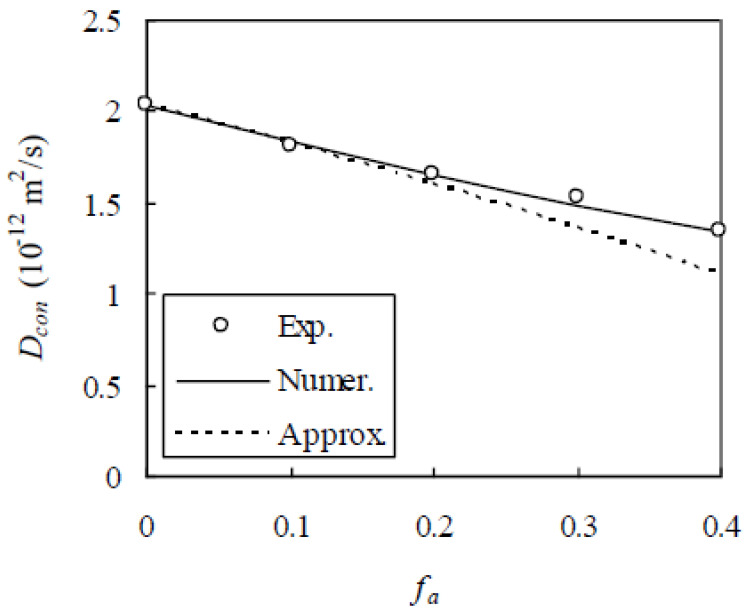
Comparison of numerical method with experimental results of Yang and Su [8] and approximate analytical solution of Shafikhani and Chidiac [38].

Further, the experimental data of Yang and Su [8] are considered. In this experiment, mortar specimens with water/cement ratio 0.4 were cast with ASTM Type I Portland cement and f_a_ was 0.0, 0.1, 0.2, 0.3, and 0.4. The aggregate volume percentage P_v,j_ passing the sieve with radius R_j_ at 0.075, 0.15, 0.30, 0.59, 1.18, 2.375, and 4.75 mm is 2.83%, 11.4%, 40.3%, 68.1%, 91.6%, 99.7%, and 100%, respectively. After 24 h of casting, the mortar specimens were demolded and cured in water at 23 °C for 12 months. A migration method was used to determine D_con_. As in the above two verification examples, h = 0.03 mm, D_bcp_ was measured as 2.03 × 10^−12^ m^2^/s, and D_i_ was calibrated as 10.2 × 10^−12^ m^2^/s from the value of D_con_ measured at f_a_ = 0.4. Thus, a comparison between the RWA and the experimental results is made as shown in Figure 16, which shows a good agreement between them. When f_a_ = 0.1, 0.2, and 0.3, the relative error between them is 1.53%, 0.520%, and 1.90%, respectively. Therefore, the validation of the RWA is preliminarily verified.

Recently, Shafikhani and Chidiac [38] derived an approximate analytical solution of the chloride diffusivity of concrete based on phenomenological multi-scale models. By considering the effects of aggregate and ITZ on the diffusion of chloride ions in concrete separately, the solution can be expressed as

(27) $$D_{\mathrm{con}}=\frac{3(1-f_{a}{)}^{2}}{3-f_{a}}\cdot \frac{12+{18\mathrm{hS}}_{a}}{12-{9\mathrm{hS}}_{a}}D_{\mathrm{bcp}}$$

where S_a_ is the aggregate surface area per unit volume of concrete and given by

(28) $$S_{a}=4\pi \langle R^{2}\rangle N_{v}f_{a}$$

With the size distribution of aggregates and D_bcp_ known, the approximate analytical solution can be calculated as shown in Figure 16. It is seen from Figure 16 that, compared with the numerical method, the approximate analytical solution underestimates the experimental results and the diviation from the experimental results increases with the increase of f_a_. The relative error between them is 0.941%, 3.09%, 10.7%, and 17.2% for a given f_a_ at 0.1, 0.2, 0.3, and 0.4, respectively. The reason for this could be that the interactions between aggregates and ITZs are not fully considered. Therefore, the numerical method developed in this paper is of higher accuracy than the approximate analytical solution.

As seen in the previous sections, there are two limitations in this study. First, to increase the computational efficiency, the EAM is adopted. As a result, the connectivity of ITZs cannot be fully embodied and the accelerated effect is neglected. Second, two of data points from each experimental database have to be chosen to calibrate the chloride diffusivities of bulk cement paste and ITZ, thereby reducing the number of data points that are used for verification. Therefore, further experimental investigations with more data points need to be conducted to provide enough statistical evidence for the validity of the RWA.

When the chloride diffusivity of concrete is determined, Fick’s second law can be used to compute the chloride profiles in concrete analytically or numerically [39,40,41] and the time for the reinforcement surface to reach the critical chloride content [42,43], which is beyond the scope of this paper.

## 6. Conclusions

Based on the RWA, a numerical method has been proposed for evaluating the chloride diffusivity of concrete. The concrete mesostructure with periodic aggregate distribution has been reconstructed for a given aggregate volume fraction and gradation and an estimated ITZ thickness. When the chloride diffusivities of two sets of concrete samples are measured, the inverse method has been used to calibrate that of ITZ. To decrease the computing cost, an EAM has been constructed. The ITZ thickness and chloride diffusivity of the equivalent aggregate have been derived. It has been shown that h_eq_/h decreases with increasing f_a_ and h and that, compared with the Fuller gradation, the EVF one has a smaller value of h_eq_/h. D_ea_/D_i_ is almost directly and inversely proportional to h_eq_ and R, respectively. With the EAM, concrete is simplified as a two-phase material. The chloride diffusivity has been estimated with the three-dimensional RWA with periodic boundary conditions on the movement of Brownian particles. The main advantage of the RWA is that there is no need to simulate the detailed zigzag walk of a Brownian particle with finite step sizes and therefore the computational time is greatly reduced and the sensitivity of chloride diffusivity to the step size is eliminated. Finally, the validation of the RWA has preliminarily been verified with experimental results.
